# Alarming levels of antimicrobial resistance among sepsis patients admitted to ICU in a tertiary care hospital in India - a case control retrospective study

**DOI:** 10.1186/s13756-018-0444-8

**Published:** 2018-12-07

**Authors:** D. Nagarjuna, Gajanand Mittal, Rakesh Singh Dhanda, Rajni Gaind, Manisha Yadav

**Affiliations:** 1 Dr. B. R. Ambedkar Centre for Biomedical Research (ACBR), University of Delhi (North Campus), Delhi, 110007 India; 2 Department of Microbiology, Vardhman Mahavir Medical College (VMMC) and Safdarjung Hospital, Delhi, 110029 India; 3 Stem Cell Laboratory, Longboat Explorers AB, SMiLE Incubator, Scheelevägen 2, 22381 Lund, Sweden; 4 Current address: Department of Biotechnology, Indian Institute of Technology, Roorkee, 247667 India; 5 Current address: Department of Clinical Microbiology, Rigshospitalet, Copenhagen, Denmark

**Keywords:** *Escherichia coli*, Sepsis, Intensive care unit (ICU), Pathotypes, Enterobacterial repeated intergenic consensus (ERIC), Antimicrobial resistance (AMR)

## Abstract

**Background:**

Hospital acquired infections (HAI) are principal threats to the patients of intensive care units. An increase in the antimicrobial resistance (AMR) observed in gram negative bacteria is a great challenge to deal with. HAI and AMR lead to prolonged hospitalization and additional doses of anti-microbial treatment affecting patient’s fitness and finances. Present study was undertaken to determine the pathotypes, genetic diversity and the antimicrobial resistance of *E.coli* in isolates from the patients admitted to intensive care unit at a tertiary care hospital in Delhi, India.

**Methods:**

*E.coli* isolates (*N* = 77) obtained from the blood culture of patients diagnosed with sepsis and the isolates (*N* = 71) from the stool culture of patients admitted in intensive care unit (ICU) but not diagnosed with sepsis were investigated for their pathotypes, adherence patterns and genetic diversity by Enterobacterial Repeated Intergenic Consensus-polymerase chain reaction (ERIC-PCR). A Kirby-Bauer Disc diffusion test and antimicrobial susceptibility assays were performed according to the Clinical and Laboratory Standards Institute (CLSI) guidelines. Extended-spectrum β-lactamase (ESBL) genes and sequence type 131 (ST131) clone were characterised genotypically by gene-specific PCRs.

**Results:**

Pathotypes analysis revealed 46 and 16% of the blood *E.coli* isolates were ETEC and EAEC respectively, in contrast to the fecal isolates wherein 22% of the isolates were ETEC and 28.5% were EAEC. EPEC, STEC and EIEC pathotypes were not detected in blood or fecal isolates. Of all the isolates studied, more than 90% of the blood and 70% of the fecal isolates were found to be resistant to cephalosporins. On the other hand, 68% of blood and 44% of the fecal isolates were found to be ESBL producers. Interestingly 83% of the blood isolates contained CTX-M15, whereas only 21% of them contained CTX-M9 genes. On the other hand CTX-M15 genes were found in 90% and CTX-M9 genes were found in 63% of the fecal isolates.

**Conclusion:**

The antimicrobial resistant profile found in this study is alarming and poses a great threat to public health. Apparently an increased antimicrobial resistance to the extensively used cephalosporins is affecting an optimal drug therapy for patients. In addition, the presence of catheters, prolonged duration of stay in the hospital and poor hygienic conditions due to infrequent urination of the patient can lead to an additional vulnerability. Therefore continuous surveillance and rational use of antibiotics along with effective hygienic measures are urgently recommended in such settings.

**Electronic supplementary material:**

The online version of this article (10.1186/s13756-018-0444-8) contains supplementary material, which is available to authorized users.

## Background

Health-care associated infections (HAIs) are a major public health concern throughout the world. Patients admitted in an intensive care unit (ICU) are at higher risk of developing bacteraemia and septicaemia [1] due to invasive procedures such as peripheral cannulation, central venous catheter placement, tracheal incubation and ventilation [2]. In addition, longer duration of stay in the hospital increases the risk of acquiring HAIs [3, 4]. The endogenous flora of the patient’s mucous membranes or hollow viscera can be the source of pathogens causing infection. Incisions made near the perineum or groin, may result in contamination with faecal flora like *Escherichia coli* (*E.coli*). Being a harmless commensal as well as a pathogen, *E.coli* exhibits great genetic diversity. It causes a wide array of disease and is responsible for around 17–37% of both community and hospital acquired clinically significant blood stream infections (BSIs) [5] and a major cause of mortality from these infections [5–8].

The rapid evolution of extended-spectrum cephalosporin and carbapenem resistance in Enterobacteriaceae which has spread globally and rapidly in the last decade is one of the most prevalent areas of drug resistance [9]. Pathogenic *E.coli* developed resistance to every class of antibiotics introduced to treat human and animal infections. Resistance to the commonly used oral antibiotics like trimethoprim-sulphamethoxazole, amoxicillin increased steadily over time. Fluoroquinolone-resistant and extended-spectrum β-lactamase (ESBL)-producing *E.coli* have enormously increased in the past two decades. The ESBL genes are frequently encoded on transferable plasmids that encode resistance genes. Acquisition of such resistant genes by commensal or fecal isolates leads to multidrug resistant (MDR) pathogens. This increase in resistance is linked to a specific clone *E.coli* sequence type 131 (ST131) that had spread worldwide since 2008 [10–15].

Previously we reported fecal *E.coli* isolates to cause endogenous infection in immune-compromised hosts. Fecal *E.coli* from the patients admitted in ICU showed similar virulence profile as that of *E.coli* isolates from the blood of sepsis patients [16]. In the present paper, we report the pathotypes, adherence patterns, genetic relatedness and the antibiotic resistance profile among blood and fecal isolates. Even though similar studies were reported [17, 18], to the best of our knowledge studies on the population at risk like those admitted in ICU were not reported from India.

## Methods

### Clinical specimens and isolation of *E.coli* isolates

A total of 148 *E.coli* isolates previously collected and studied for their virulence profile and phylogroups [16] were used for this study. Samples were collected from February 2011 to August 2013 as described before [16]. Briefly, the first group of *E.coli* isolates (*N* = 77) were obtained from the blood culture of sepsis patients and the second group (*N* = 71) were obtained from the faeces of patients who were admitted in ICU for various reasons for example had undergone cardiovascular surgery, cases of road transport accident etc. but were not diagnosed with sepsis.

### *E.coli* Pathotypes

Pathotyping was performed by a multiplex PCR using the primers *uidA****,*** *pic, bfp, invE, LT, escV, aggR, stx1a, stx2a, st1b, st1a, astA* corresponding to the genes defining the appropriate pathotypes as previously reported [19]. EAEC strains harbour *astA*, *aggR*, and *pic* genes and can be confirmed if found positive in a combination of *pic and aggR* or *aggR* and *astA.* Isolates found positive for either LT toxin (LT) or heat-stable toxin (ST) were designated as ETEC, whereas designated as EIEC if found positive for *invE*, and as EPEC if found positive for *escV and bfp*. STEC isolates were positive for *stx* and negative to *bfp* however in the presence or absence of *escV*. A primer pair for the detection of the *E.coli*-specific *uidA* gene was also included.

### HeLa cells culture & adherence assays

HeLa cells were grown in Dulbecco’s Modified Eagle Medium (DMEM) with 10% fetal bovine serum (FBS; Pan-Biotech, Germany) in the presence of 1% antibiotic mixture (penicillin and streptomycin; Life Technologies, USA) in an environment of 5% CO_2_ at 37 °C. Adherence assay was performed on the monolayer of HeLa cells, which were upto 50% confluent [18]. Briefly, HeLa cells were first washed with phosphate buffered saline (PBS, pH 7.4). After washing, 1.0 ml of fresh medium (DMEM supplemented with 2% FBS) was added to the cell monolayers. The HeLa cells thereafter were inoculated with approximately 10^8^ CFU/mL suspensions of *E.coli* grown in LB broth (overnight culture), diluted 1:50 and incubated at 37 °C. After 6 h of incubation, the cells were washed twice with PBS and thereafter fixed with methanol (Merck, Germany) for 1 h. The methanol-fixed cells were then stained with May Grünwald-Giemsa stain for 1 h and destained with 70% ethanol. Cells were observed under inverted microscope after fixation at 20X (Nikon, Eclipse TS100).

### Analysis of Enterobacterial repeated intergenic consensus (ERIC) sequences

*E.coli* isolates were fingerprinted using ERIC-PCR. The primers used for the ERIC-PCR reaction were ERIC-F 5’-AAGTAAGTGACTGGGGTGAGCG-3’ and ERIC-R 5’-ATGTAAGCTCCTGGGGATTCAC-3′ [20]. The gel images were captured using a Gel-documentation system. All the bands obtained were normalized using ImageLab software. Depending upon the molecular weight of the reference marker, a weighted matrix was generated. Using the PyELph v1.4 software, each band in each lane was analysed to obtain the band size with reference to the marker bands. All the band sizes so obtained were treated as an input for further analysis. A binary code of 1 or 0 was introduced to each band subjected to the presence and/or absence of the band respectively. On the basis of such generated binary matrix file, a phylogenetic tree and principle component analysis was constructed through NTSYS-pc 2.02 J software. Band intensity is an important characteristic for this analysis. The bands with very low resolutions were ignored by the ImageLab software as background noise after manually checking each band. The band profiles of the DNA fragments obtained after PCR amplification using specific primers for ERIC sequences were determined. The fingerprints obtained consisted of 5 to 15 bands ranging in size from 100 bp to 1 kb.

### Antibiotic susceptibility assay

Individual antimicrobial disks of Amikacin 30mcg (AK), Cefepime 30mcg (CPM), Cefoperazone 75mcg (CPZ), Cefoxitin 30mcg (CX), Ceftazidime 30mcg (CAZ), Ciprofloxacin 5mcg (CIP), Gentamicin 10mcg (GEN) (HiMedia, India) were placed on the surface of the agar using sterile forceps. The disks were in complete contact with the agar surface by pressing down with forceps. 10 disks each were placed on a 150-mm plate and each had a gap of more than 24 mm between them. The plates were thereafter inverted and incubated at 37 °C for 18 h. Diameters of the inhibition zones were measured to the nearest millimetre using calibrated scale.

### Screening of ESBL producers

An inert flat circular ring having a disk of Aztreonam (30 μg), Cefpodoxime (10 μg), Cefpodoxime/Clavulanic acid (10/5 μg), and Ceftazidime (30 μg) with a 6 mm diameter on its projections was used. According to the CLSI guidelines [21], isolates showing Cefpodoxime(10 μg) < 17 mm, Ceftazidime(30 μg) < 22 mm, Aztreonam (30 μg) < 27 mm, Cefotaxime (30 μg) < 27 mm, Ceftriaxone (30 μg) < 25 mm in the initial screening were considered as potential ESBL-producer. ESBL producer isolates were further screened with another set of discs having Cefpodoxime (10 μg), Cefpodoxime/Clavulanic acid (10/5 μg), Ceftazidime (30 μg), Ceftazidime/Clavulanic acid (30/10 μg), Cefotaxime (30 μg) and Cefotaxime/Clavulanic acid (30/10 μg). An increase of ≥2 mm in zone diameter for antimicrobial agent that were tested alone versus when tested in combination with Clavulanic acid confirmed the isolate as a potent ESBL producer.

### Genotypic characterisation of ESBL genes

The presence of ESBL genes was tested by two multiplex PCRs, the first one detects TEM/SHV/ OXA-1 (Temoneira/ Sulfhydryl variable/ Oxacillin hydrolysing capabilities) group and the second one detects CTX-M groups 1, 2 and 9 [22].

### Detection of ST131 gene and Fim H30 and H30-Rx sub-clones by PCR

Detection of the ST131-O16 and ST131-O25b clades was carried out by PCR using the primers previously described [23]. ST131 isolates were further characterized by screening them for ST131-associated SNPs in *mdh* (i.e., C288T and C525T) and *gyrB* (i.e., C621T, C729T, and T735C) [24]. Further, ST131-associated Fim H30 and H30-Rx subclone were identified by PCR. All ST131 positive isolates were tested for *fimH* 30 allele (encoding a variant of the type 1 fimbrial adhesin) corresponding with the main FQ resistance–associated subset within ST131, using the allele-specific primers as decribed before [24, 25]. The H30-Rx sub-clone was identified by detection of a specific single-nucleotide polymorphism (SNP) (G723A) within the allantoin-encoding gene, ybbW using the Primers AP63 and AP66 as described before [26].

### Statistical analysis

Fishers Exact test was used to compare pathotypes between the blood and fecal *E.coli* isolates. Z-test was used to compare the prevalence of ESBL and ST131 clone between the blood and fecal *E.coli* isolates. *P* value of *p* < 0.05 was considered significant.

## Results

### Comparison of pathotypes between blood and fecal *E.coli* isolates

**Fig. 1:**
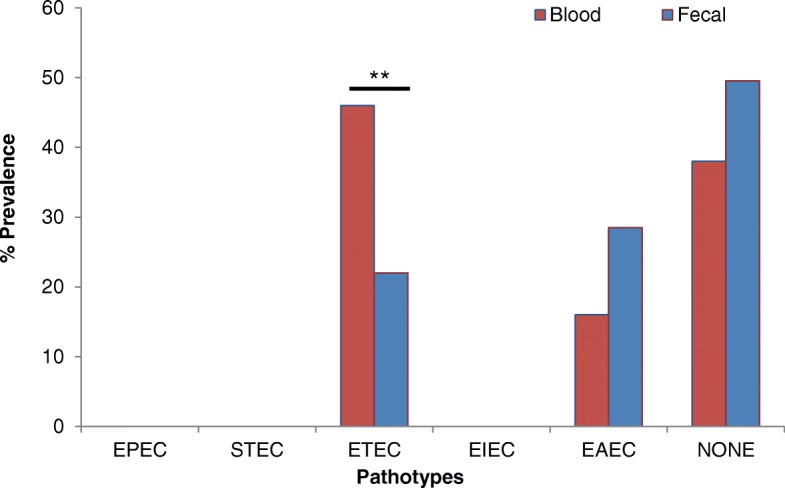
Prevalence of pathotypes in blood and fecal *E.coli* isolates from sepsis and non-sepsis patients respectively. *p* value is **p ≤ 0.01. EPEC: Enteropathogenic *E.coli;* STEC: Shiga toxin-producing *E.coli*; ETEC: Entero-toxigenic *E. coli;* EIEC: entero-invasive *E.coli*; EAEC: Entero-aggregative *E.coli*

Our data showed that 62% of the blood *E.coli* isolates were designated to one of the pathotypes studied, among which 46% were ETEC and 16% were EAEC. While in case of fecal *E.coli* isolates (50.5%), 22% were ETEC and 28.5% were EAEC ((Fig. 1). 38% of the blood and 49.5% of the fecal isolates do not belong to any of the pathotypes investigated (Fig. 1). We did not find any of the blood or fecal *E.coli* isolates positive for EPEC, STEC and EIEC.

### Adherence assays in HeLa cells

**Fig. 2:**
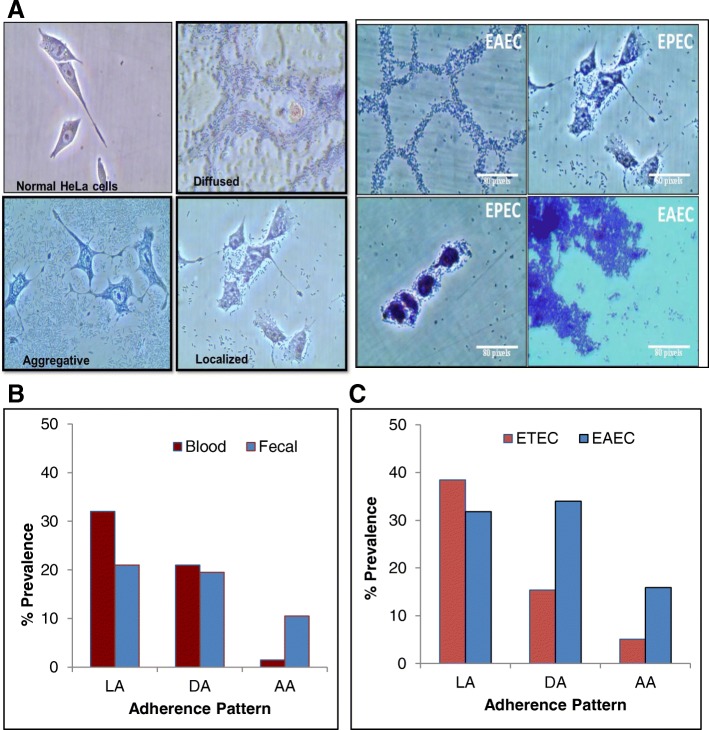
**a** Representative characteristic adherence patterns of *E.coli* in EAEC and ETEC as seen in HeLa cells upon infection (L: Localized; D: Diffused and A: Aggregative pattern). Scale is 100 μM; **b** Overall prevalence of adherence patterns in blood and fecal *E.coli* isolates from sepsis and non-sepsis patients respectively; **c** Prevalence of adherence patterns among EAEC and ETEC

Localized adherence was observed among 32 and 21% of the blood and fecal isolates. Diffused adherence pattern was observed in 20% of both blood and fecal isolates. Aggregative adherence was observed among 10.5% of the fecal and only 1.5% of the blood isolates (Fig. 2a, b). A large proportion of the isolates showed localized adherence, which is characteristic of EPEC, but none of the isolates were found positive for *bfp*. Among the isolates designated as ETEC based on the PCR results, some of the isolates were locally adhered (38.46%) some were diffused (15.38%) and a few with aggregative adherence (5.1%). Among the isolates designated as EAEC, a large proportion showed diffused adherence (31.81%) and aggregative (15.9%) thereby correlating with the PCR results. (Fig. 2a, c).

### ERIC analysis

**Fig. 3:**
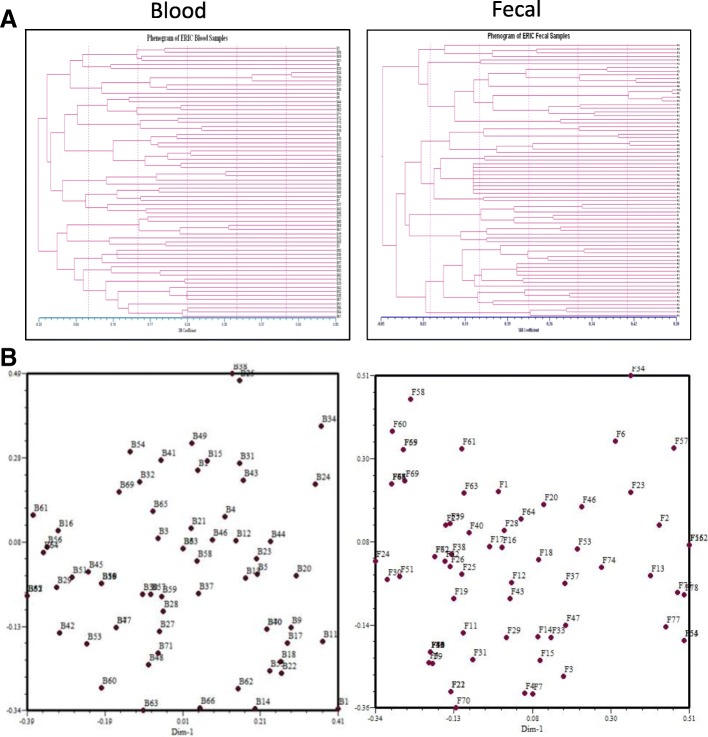
**a** Phenogram and **b** Principal component analysis showing the genomic similarity among blood and the fecal *E.coli* isolates as carried out by computer-assisted ERIC-PCR DNA fingerprint analysis (see material and methods for detail)

The ERIC–PCR profiles allowed differentiation of all the *E.coli* isolates into six main clusters. Both analyses were reported under ~ 0.00 to 0.50 matrix distance evaluation. All the blood isolates analysed were found to be clustered into two groups at 0.25 SM with Cluster 1 comprising of 44 *E.coli* isolates and cluster 2 comprising 22 *E.coli* isolates (Fig. 3a). The fecal *E.coli* isolates were clustered into four groups at 0.25 SM with cluster 1 comprising of 22 *E.coli* isolates, followed by cluster 2 of 21 *E.coli* isolates, cluster 3 of 20 *E.coli* isolates and cluster 4 comprising of 11 *E.coli* isolates (Fig. 3a). We observed that blood isolates were more similar with respect to the banding pattern. The principal component analysis (PCA) showed the diversity among the blood and fecal *E.coli* isolates. The fecal *E.coli* isolates were found to be more diverse as observed by PCA analysis (Fig. 3b).

### Antibiotic susceptibility assay

**Fig. 4:**
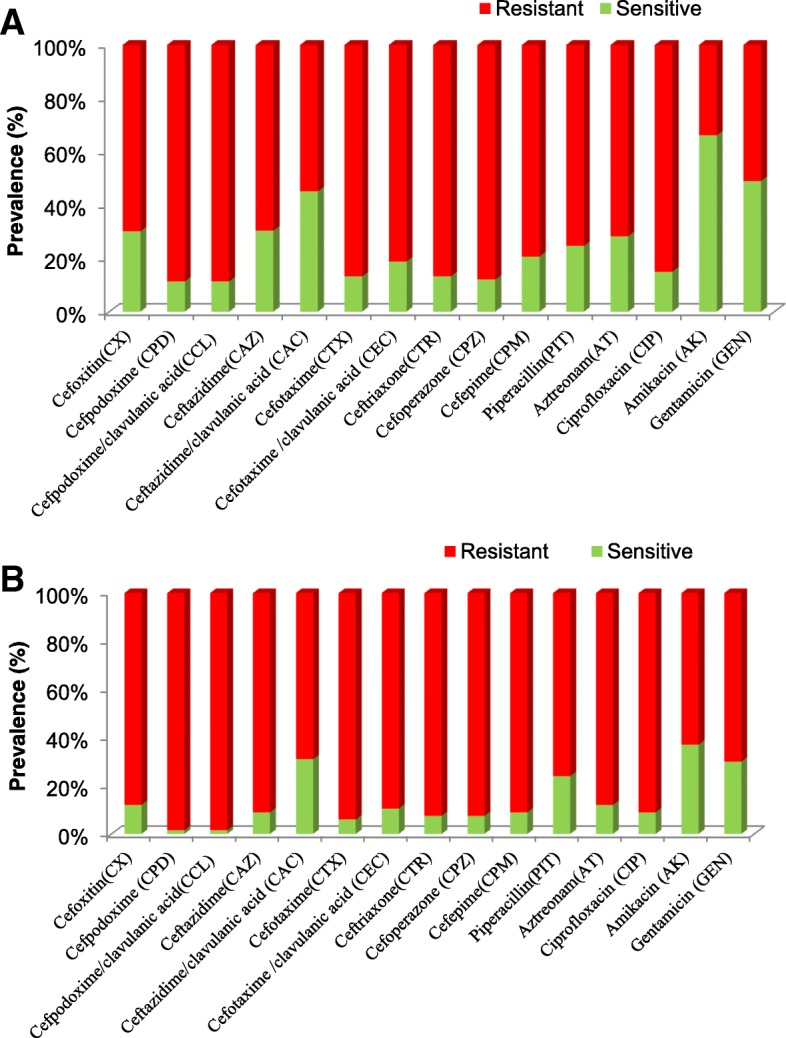
Antibiotic susceptibility pattern of fecal (**a**) and blood (**b**) *E.coli* isolates from non-sepsis and sepsis patients respectively

The fecal *E.coli* isolates were found to be resistant for Cefoxitin (70%), Cefpodxime (89%), Cefpodoxime/clavulanic acid (89%), Ceftazidime (69%), Ceftazidime/clavulanic acid (55%), Cefotaxime (87%), Cefotaxime/Clavulanic acid (81%), Ceftriaxone (87%), Cefaperazone (88%) and Cefipime (79%) (Fig. 4a). Among the blood *E.coli* isolates, 88% were found to be resistant for Cefoxitin, Cefpodxime (98.5%), Cefpodoxime/clavulanic acid (98.5%), Ceftazidime (91%), Ceftazidime/clavulanic acid (69%), Cefotaxime (94%), Cefotaxime/clavulanic acid (90%), Ceftriaxone (92.5%), Cefaperazone (92.5%) and Cefepime (91%) (Fig. 4b). Fecal *E.coli* isolates were resistant to Piperacillin (75%), Aztreonam (72%), Amikacin (34%), Gentamicin (51%), Ciprofloxacin (85%). Whereas blood *E.coli* isolates were found to be resistant for Piperacillin (76%), Amikacin (63%), Gentamicin (70%), Ciprofloxacin (91%), and Aztreonam (88%) (Fig. 4a-b).

### ESBL producers and identification of ESBL genes

**Fig. 5:**
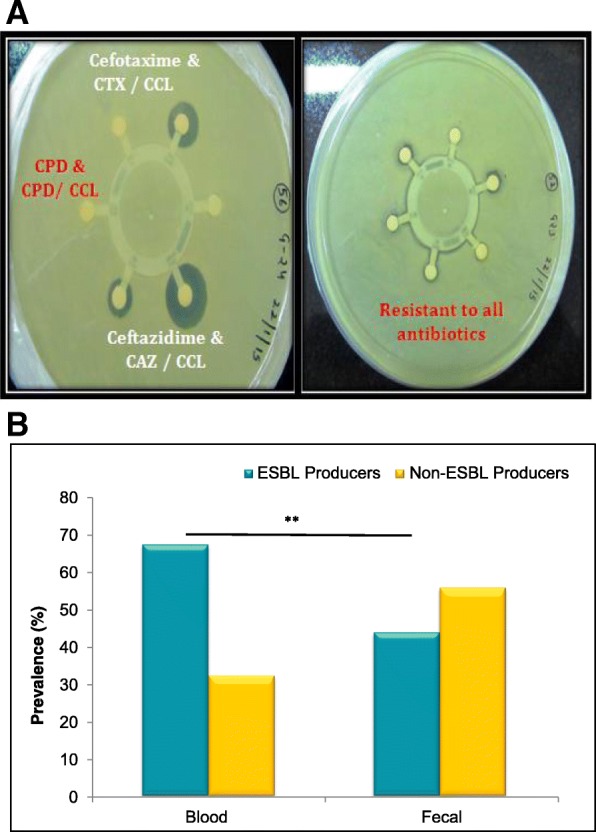
**a** Picture representing the antibiotic disc diffusion test for extended spectrum Beta-Lactamase (ESBL) producers. Each circular disc contains the antibiotic of specific concentration. Clear zone around the disc indicates zone of Inhibition. **b** Prevalence of ESBL producers between blood and fecal *E.coli* isolates from sepsis and non-sepsis patients respectively. *P* value is **p = 0.001

**Fig. 6:**
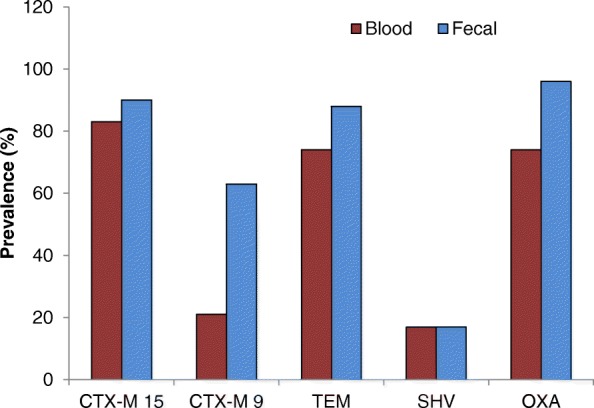
Comparision of different ESBL genes in blood and fecal *E.coli* isolates from sepsis and non-sepsis patients respectively using gene specific PCR

ESBL producers confer resistance to third generation Cephalosporins (e.g., Ceftazidime, Cefotaxime, and Ceftriaxone) and monobactams (e.g., Aztreonam) but do not affect cephamycins (e.g., Cephoxitin and Cefotetan) or carbapenems (e.g., Meropenem or Imipenem) (Fig. 5a). According to the observed susceptibility patterns, it was found that 68% of blood *E.coli* isolates were found to be ESBL producers whereas 44% of the fecal isolates were confirmed as ESBL producers (Fig. 5b). ESBL genes were identified by ESBL gene specific PCR. Among the blood *E.coli* isolates, 83% of the isolates showed CTX-M15, while only 21% of them had CTX-M9, whereas TEM was observed in 74%, SHV in 17%, OXA-1 in 74% of the isolates. In case of fecal *E.coli* isolates, CTX-M15 was observed in 90%, CTX-M9 in 63%, of the isolates and TEM, SHV, and OXA-1 were observed in 88, 17, 96% respectively (Fig. 6).

### Prevalence of ST131 clone

**Fig. 7:**
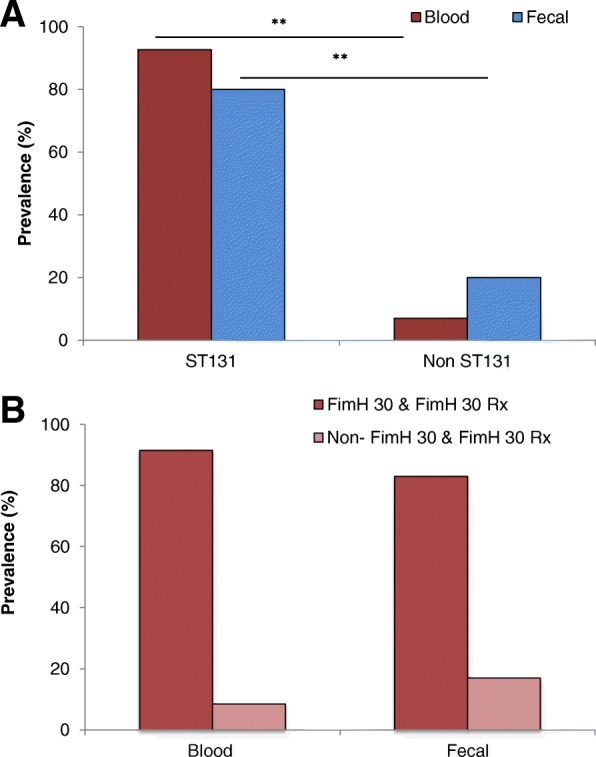
**a** Prevalence of ST131 clone **b** Prevalence of FimH30 and FimH30-Rx sub groups of ST131 clone among blood and fecal *E.coli* isolates from sepsis and non-sepsis patients respectively. **p = 0.0119

ST131 clone was identified using ST131 clone specific PCR method (Additional file 1: Figure S1). Our results showed that 80% of the fecal *E.coli* isolates were positive for ST131 clone while in 92% among the blood isolates (Fig. 7a). In addition, all the *E.coli* isolates were further used for identification of ST131 with associated SNPs i.e. *mdh*36 and *gyrB*47. We found that all ST131 positive isolates were also positive for both ST131 associated SNPs. All the isolates that were positive for ST131 were tested and further confirmed for FimH 30 and FimH 30-Rx sub groups of ST131 clone (Additional file 2: Figure S2). We found that 91.5% of the blood *E.coli* isolates and 83% of the fecal isolates belong to the FimH 30 and FimH 30-Rx sub groups (Fig. 7b).

## Discussion

Identification of *E. coli* pathotypes in association with blood stream infections is limited in many developing countries because routine diagnostic screens only the micro-organism, the conventional microbiological testing is unable to distinguish between normal flora and pathogenic strains of *E. coli* [27]. Entero pathogenic *E.coli* (EPEC) is a major etiological agent of infant diarrhea predominantly in developing countries [28–30]. ETEC defined by their production of the plasmid-encoded heat-labile (LT) and heat-stable (STIa/STIb) toxins is the leading cause of traveler’s diarrhea [31]. ETEC & EAEC are the most commonly identified pathogens in our study. It was found that the proportion of ETEC was significantly higher among the blood isolates as compared to fecal *E.coli* isolates (*p* = 0.029). However, we did not find any significant difference in the proportion of EAEC between blood and fecal *E.coli* isolates. Our data showed a large proportion of the isolates with localized adherence, which is a known characteristic of EPEC however none of the isolates were found positive for EPEC by PCR.

All the blood isolates analysed by ERIC-PCR were found to be clustered into two groups while fecal *E.coli* isolates were clustered into four groups. The principal component analysis (PCA) of the blood *E.coli* isolates were more similar among themselves with respect to the ERIC band profiles while the fecal *E.coli* isolates were more diverse. We can correlate the observation of PCA and cluster analysis with the hypothesis that a single strain from the gut may be the source of endogenous infection which may prompt an “overspill” bacteraemia.

Antibiotic susceptibility results showed that more than 70% of the fecal *E.coli* isolates and more than 90% of the blood isolates were resistant to all of the cephalosporins tested. Among the fecal isolates, we observed a slight decrease in susceptibility to cephalosporin in combination with an inhibitor clavulanic acid. Fecal *E.coli* isolates were resistant to Cefpodoxime/clavulanic acid (89%), Ceftazidime/clavulanic acid (55%) and Cefotaxime/clavulanic acid (81%). While blood *E.coli* isolates were resistant to Cefpodoxime/clavulanic acid (98.5%), Cefotaxime/clavulanic acid (90%), Ceftazidime (91%) and Ceftazidime/clavulanic acid (69%). A significant decrease in susceptibility to Ceftazidime in combination with clavulanic acid was observed as compared to Ceftazidime alone.

However, among the other classes of antibiotics aminoglycosides, fluoroquinolone, and monobactams studied, 85% of the fecal isolates were resistant to Ciprofloxacin, 75% were resistant to Piperacillin, 72% for Aztreonam, 34% for Amikacin and 51% for Gentamicin. Whereas, 91% of the blood isolates were resistant to Ciprofloxacin, 76% were resistant to Piperacillin, 88% for Aztreonam, 63% for Amikacin, and 70% for Gentamicin. In comparison to blood isolates, fecal isolates were more susceptible to amikacin (34% verses 63%) and gentamicin (51% verses 70%). Overall, 68% of the blood isolates were found to be ESBL producers whereas 44% of the fecal isolates were confirmed as ESBL producers by observing the susceptibility patterns in disc synergy tests with clavulanic acid.

Antibiotic resistance in *E.coli* can be conferred by both chromosomal and plasmid-encoded genes. Resistance to ciprofloxacin was observed in concurrence with cephalosporin resistance. ESBLs degrade the β-lactam moiety of penicillin derivatives, cephalosporins, monobactams, and Carbapenems. The ESBL genes are frequently encoded on transferable plasmids that encode resistance genes. Acquisition of such resistant genes by commensal or fecal isolates leads to MDR pathogens. The three major groups of ESBL enzymes are TEM, SHV and CTX-M. Among the CTX-M-type ESBLs, CTX-M-15 is widely distributed worldwide [32], and are the most prevalent in India [33]. In our study, we found that 83% of the blood *E.coli* isolates whereas 90% of the fecal isolates showed CTX-M15; CTXM-15 producing isolates were reported to have reduced susceptibility to Cefepime [34]. Our results are in line with the observation since 91 and 79% of the blood and fecal isolates respectively are resistant to Cefepime. Therefore, Cefepime which is a fourth generation cephalosporin is administered intravenously and used primarily for treatment of pneumonia, UTIs, and intra-abdominal infections, can no longer be a choice of drug.

The gut has been thought to be a repository of pathogens and an incredible source for the development of antibiotic resistance [35]. Overall the carriage of ESBL genes is more in fecal isolates than that of the blood isolates CTX-M9 (63% verses 21%), CTX-M15 (90% verses 83%), TEM (88% verses 74%), and OXA-1 (96% verses 74%). Previously, it was demonstrated as an intestinal colonization by gram-negative organisms before the onset of the disease [36]. We propose a similar scenario since we have found CTX-M15 as a predominant ESBL gene among the fecal isolates in our study. There is a significant increase in the prevalence of CTX-M enzyme producing *E.coli* worldwide. We report the detection of CTX-M group 9 genes and the CTX-M15 as a predominant ESBL gene among fecal isolates. Our results also highlight the importance of studying gut flora in assessing the changing repertoire of organisms to investigate the pattern of antibiotic susceptibilities. We found prevalence of ESBL-producers more among the blood isolates whereas the isolates showing the ESBL genes were found predominantly among fecal isolates.

The high resistance patterns against all of the 15 antibiotics we studied compelled us to further analyse the isolates for the ST131 clone. *E.coli* ST131 clone is well known affiliated with the worldwide spread of CTX-M15 enzyme. ST131 isolates were associated with extra-intestinal infections, frequently in UTI and bacteraemia. Initially detected in a community, later ST131 isolates were also obtained from health care settings [13]. ST131 strains are MDR and patients with such infections are at high risk of having constrained treatment choices with a prolonged duration of disease. We observed that 80% of the fecal isolates and 92% of the blood isolates belonged to ST131 clone, a sub-clonal lineage of *E.coli* ST131 that contains the type 1 fimbriae fimH30 (H30) allele and is termed as FimH 30 sub group. Isolates of this sub group were reported resistant to fluoro-quinolones (FQ) with only < 1% of FQ-susceptible isolates. The abrupt expansion and genetic similarity among the H30 strains clue that the emergence of FQ-resistant ST131 strains was driven by clonal expansion and dissemination. Isolates of H30 ST131 sub-clone were found to be resistant for more than 3 antibiotic classes and with CTX-M1. Within the H30 lineage, Price et al., identified a close distinct sub lineage with more extensive antimicrobial resistance profile called H30-Rx. This sub lineage was formed from H30 strains that carried CTX-M15 distinguished from ESBL-negative H30 strains by 3 core genome SNPs [26]. Interestingly, in association with the high resistance to Ciprofloxacin (85% fecal and 91% blood isolates), we found 83% of the fecal and 91.5% of the blood *E.coli* isolates belonged to FimH 30 and FimH 30-Rx sub groups.

A few studies reported the prevalence of ST131 clone from India. Among them is a study from neonatal isolates, which reported 9% prevalence of ST131 [37]. Another study reported 70% prevalence of ST131 among the ESBL producing strains [38]. MLST is the most accurate and competent method for detection of ST131 clones. But it is tedious and costly. Especially, its application for investigating the MDR clone for clinical diagnosis is not possible. ST131 clone rapid detection assays were previously reported [23, 39]. Even though those methods may validate ST131, the results usually vary and need a confirmation by MLST. It is alarming to find a higher prevalence of ST131 clone isolates in our study evidenced by extreme antibiotic resistant and carriage of ESBL genes.

## Conclusion

This study emphasizes the need for the differential identification of specific pathotypes in order to facilitate appropriate counter measures. The genomic diversity analysed by ERIC-PCR portrayed the diversity of *E.coli* strains in the gut. There are more outliers among the fecal isolates. A large extent of blood isolates studied were ESBL-producers and resistant to Cephalosporins. Even though the resistant profiles of fecal isolates are lower in comparison to blood isolates, the isolates lodging the antibiotic resistant genes were more among the fecal isolates. ST131 strains are MDR and linked to spread of the antibiotic resistance. We observed a higher prevalence of ST131 isolates. Patients with such infections are at risk of constrained treatment choices and prolonged duration of disease. The antimicrobial resistance profiles found in this study pose a great threat to public health. Increasing anti-microbial resistance among *E.coli* to the commonly used cephalosporins hinders the decision of the optimal drug therapy for patients. Continuous surveillance and rational use of antibiotics along with effective hygienic measures are urgently needed in our setting.

### Study bias and weakness/limitations

There may be selection bias as a fraction of cases were selected for the study from a large group of patients in the ICU. The controls samples may be more biased as they were selected based on a few inclusive criteria that they are admitted in ICU and not having sepsis.

## Additional files

Additional file 1: **Figure S1.** PCR amplified product of *pabB* and *uidA* run in 1.8% agarose gel. Lane 1–12: Samples, Lane 11: positive control, Lane 12: Negative control, Lane 13: 100 bp Ladder, Lane 1–6 and 10 shows presence of *pabB* (347 bp) & *uidA* (657 bp) indicating ST131, Lane 7 and 9 were negative for ST131. (ODP 188 kb)

Additional file 2: **Figure S2.** PCR amplified product of FimH 30 and FimH30 Rx sub groups run on 1.8% agarose gel. Lane 1–10: Samples, Lane 11: 100 bp Ladder, Lane 1–10 except 2 shows bands at 194 bp & 354 bp indicating FimH 30 and FimH 30 Rx sub groups. (ODP 119 kb)

## Abbreviations

AMR: Antimicrobial resistance
CLSI: Clinical and laboratory standards institute
DMEM: Dulbecco’s modification of eagle medium
ERIC: Enterobacterial repeated intergenic consensus
ESBL: Extended-spectrum β-lactamase
HAI: Hospital acquired infections
ICU: Intensive care unit
OXA: Oxacillin hydrolysing capabilities
PCA: Principal component analysis
PCR: Polymerase chain reaction
SHV: Sulfhydryl variable
ST131: Sequence type 131
TEM: Temoneira
