# Supplementary figures and images for: Alarming levels of antimicrobial resistance among sepsis patients admitted to ICU in a tertiary care hospital in India - a case control retrospective study
Source: Antimicrob Resist Infect Control. 2018 Dec 7;7:150. doi: 10.1186/s13756-018-0444-8 (PMC6286518; doi:10.1186/s13756-018-0444-8)

## Slide: page1
Figure S1
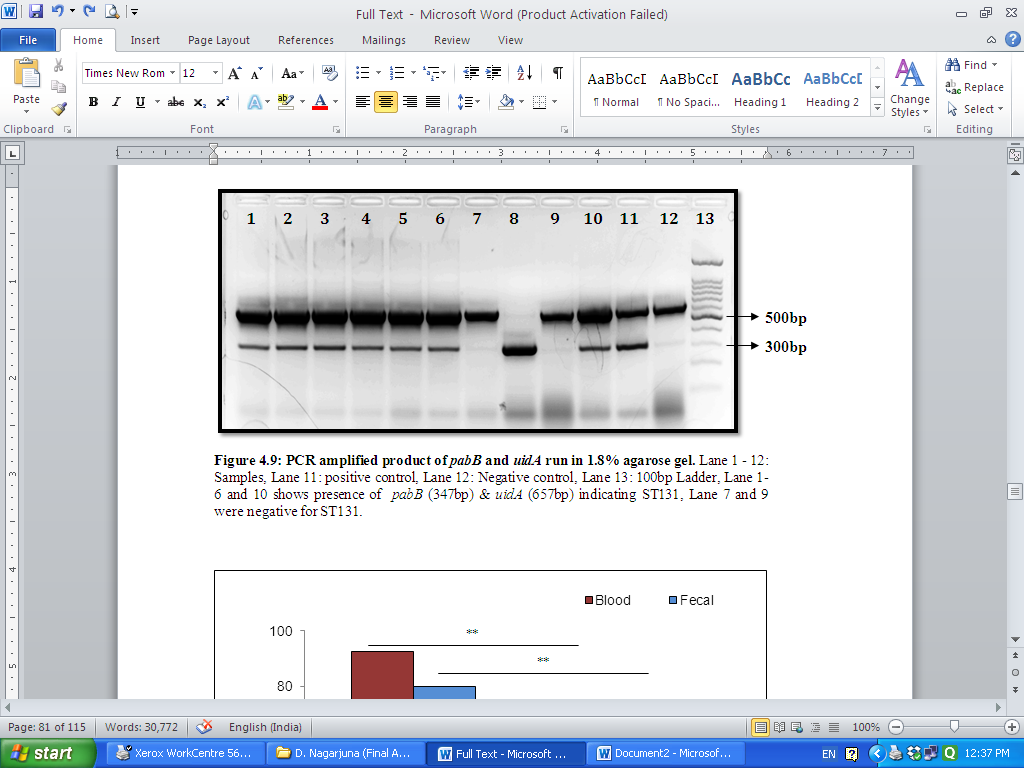

500 bp
300 bp

Supplement: Supplementary file 1 — Figure S1. PCR amplified product of pabB and uidA run in 1.8% agarose gel. Lane 1–12: Samples, Lane 11: positive control, Lane 12: Negative control, Lane 13: 100 bp Ladder, Lane 1–6 and 10 shows presence of pabB (347 bp) & uidA (657 bp) indicating ST131, Lane 7 and 9 were negative for ST131. (ODP 188 kb) [file 13756_2018_444_MOESM1_ESM.odp]

## Slide: page1
Figure S2
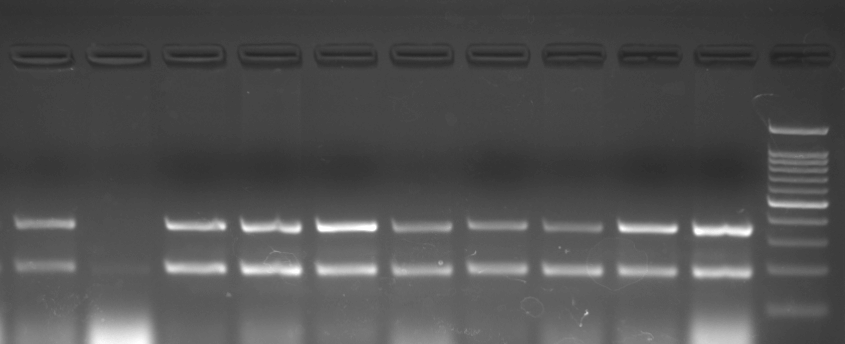

 1  2  3  4  5  6  7  8  9  10  11


200bp
300bp
400bp

Supplement: Supplementary file 2 — Figure S2. PCR amplified product of FimH 30 and FimH30 Rx sub groups run on 1.8% agarose gel. Lane 1–10: Samples, Lane 11: 100 bp Ladder, Lane 1–10 except 2 shows bands at 194 bp & 354 bp indicating FimH 30 and FimH 30 Rx sub groups. (ODP 119 kb) [file 13756_2018_444_MOESM2_ESM.odp]
